# Critical switching current density of magnetic tunnel junction with shape perpendicular magnetic anisotropy through the combination of spin-transfer and spin-orbit torques

**DOI:** 10.1038/s41598-021-02185-3

**Published:** 2021-11-24

**Authors:** Doo Hyung Kang, Mincheol Shin

**Affiliations:** School of Electrical Engineering, Korea Advanced Institute of Science and Technology, Daejeon, 34141 South Korea

**Keywords:** Physics, Condensed-matter physics, Magnetic properties and materials

## Abstract

Recently, magnetic tunnel junctions (MTJs) with shape perpendicular magnetic anisotropy (S-PMA) have been studied extensively because they ensure high thermal stability at junctions smaller than 20 nm. Furthermore, spin-transfer torque (STT) and spin-orbit torque (SOT) hybrid switching, which guarantees fast magnetization switching and deterministic switching, has recently been achieved in experiments. In this study, the critical switching current density of the MTJ with S-PMA through the interplay of STT and SOT was investigated using theoretical and numerical methods. As the current density inducing SOT ($J_{\text {SOT}}$) increases, the critical switching current density inducing STT ($J_{\text {STT,c}}$) decreases. Furthermore, for a given $J_{\text {SOT}}$, $J_{\text {STT,c}}$ increases with increasing thickness, whereas $J_{\text {STT,c}}$ decreases as the diameter increases. Moreover, $J_{\text {STT,c}}$ in the plane of thickness and spin-orbit field-like torque ($\beta$) was investigated for a fixed $J_{\text {SOT}}$ and diameter. Although $J_{\text {STT,c}}$ decreases with increasing $\beta$, $J_{\text {STT,c}}$ slowly increases with increasing thickness and increasing $\beta$. The power consumption was investigated as a function of thickness and diameter at the critical switching current density. Experimental confirmation of these results using existing experimental techniques is anticipated.

## Introduction

Since the pioneering research of Slonczewki and Berger^1,2^, spin-transfer torque (STT) has been studied experimentally and theoretically because it has a high practical potential for spin torque nano-oscillators (STNOs)^3–8^ and STT magnetic random access memory (STT-MRAM)^9–14^, as well as academic research. At the STT-MRAM point, the magnetic tunnel junction (MTJ), the core cell of the MRAM, must maintain a lower switching current density to attain low power, high thermal stability to obtain high reliability, and a small size to achieve higher-density integration simultaneously^15–17^. Furthermore, a lower current density can prevent the tunnel barrier breakage. Since MTJ with interfacial perpendicular magnetic anisotropy (I-PMA) guarantees low current density and high thermal stability, the study of MTJ with I-PMA is replacing that of MTJ with in-plane magnetic anisotropy (IMA)^18–21^. STT-MRAM using MTJ with I-PMA has been commercialised^22,23^. However, owing to the decrease in thermal stability below 20 nm in diameter, the MTJ with I-PMA has the limitation in size. The thermal stability is approximately $40{-}50$ in the range of $10{-}15$ nm in diameter^23^. Therefore, the MTJ with shape perpendicular magnetic anisotropy (S-PMA) should be considered for high thermal stability below 20 nm.

Recently, for the MTJ with S-PMA, thermal stability and current-induced magnetization switching have been studied by experiments and simulation^17,23–26^. The thermal stability shows various distributions depending on diameter and thickness below 20 nm by combining the demagnetization energy and I-PMA energy. For example, in the case of $D=10{-}15$ nm, when $t_f\ge 20$ nm, the thermal stability is more than 120. When the *D* is $5{-}10$ nm, the thermal stability is $40{-}80$, where $t_{\text {f}}$ and *D* are the thickness and diameter of the free layer, respectively^23^. Magnetization switching using STT is achieved with a TMR of 20–100% for $t_{f}=60$ nm and various diameters ($5{-}30$ nm) in the case of the Co storage layer, whereas the FeB blanket film storage layer exhibits a TMR of 100% for $t_{f}=15$ nm and $D=8, 10$, and 15 nm. However, the critical current density required for magnetization switching is $3.9\times 10^{11}$ $\text {A/m}^{2}$, which can damage the tunnel barrier. In addition, the switching time delay caused by the incubation time does not allow magnetization reversal in picoseconds.

Magnetization switching of the MTJ with perpendicular magnetic anisotropy induced by spin-orbit torque (SOT) is achieved with a switching time in picoseconds and without damage to the MgO tunnel barrier^27–35^. The SOT generated by the in-plane spin current originating from the spin Hall effect or the Rashiba spin-orbit effect^36–38^ does not guarantee the deterministic switching. An external magnetic field is required for deterministic switching. Magnetization switching of perpendicularly magnetized MTJ through the interplay of STT and SOT makes deterministic switching possible without an external magnetic field, along with methods of inducing lateral inversion symmetry breaking through the lateral structural asymmetry, generating the exchange field using an antiferromagnetic layer and inducing symmetry breaking through the tilted magnetic easy axis^30,32,34,39,40^. STT-SOT switching of the MTJ with I-PMA has been studied through experiments and micromagnetic simulations^41–43^. The current density for magnetization switching decreased as the SOT current density increased. In addition, the incubation time of STT-SOT switching was greatly reduced in SOT-dominant switching^41^. In terms of power consumption, the energy efficiency is improved compared with STT switching, and the smaller the radius of the MTJ, the lower the power consumption^42^. Compared with SOT switching with an external magnetic field, STT-SOT switching significantly improves deterministic switching, even when the MTJ is deformed^43^. As with STT-SOT switching of MTJ with I-PMA, one can expect a fast magnetization switching time in the STT-SOT switching of the MTJ with S-PMA.

In this study, the critical switching current density of the MTJ with S-PMA through the interplay of STT and SOT was investigated by employing an analytical study and macrospin simulation, where the analytical formula were derived using the linearized Landau-Lifshitz-Gilbert (LLG) equation in the rotation coordinate. The analytical estimation results for the dependence of the STT-induced critical switching current density, $J_{\text {STT,c}}$, on the SOT-induced critical switching current density, $J_{\text {SOT,c}}$, are consistent with the numerical calculation results. The thickness, diameter, and spin-orbit field-like torque dependence of $J_{\text {STT,c}}$ was also investigated, providing a design rule for STT-SOT MRAM based on the MTJ with S-PMA. Furthermore, the power consumption by the STT for a given $J_{\text {SOT}}$ as a function of thickness and diameter was investigated.

## Analytical investigation

**Figure 1:**
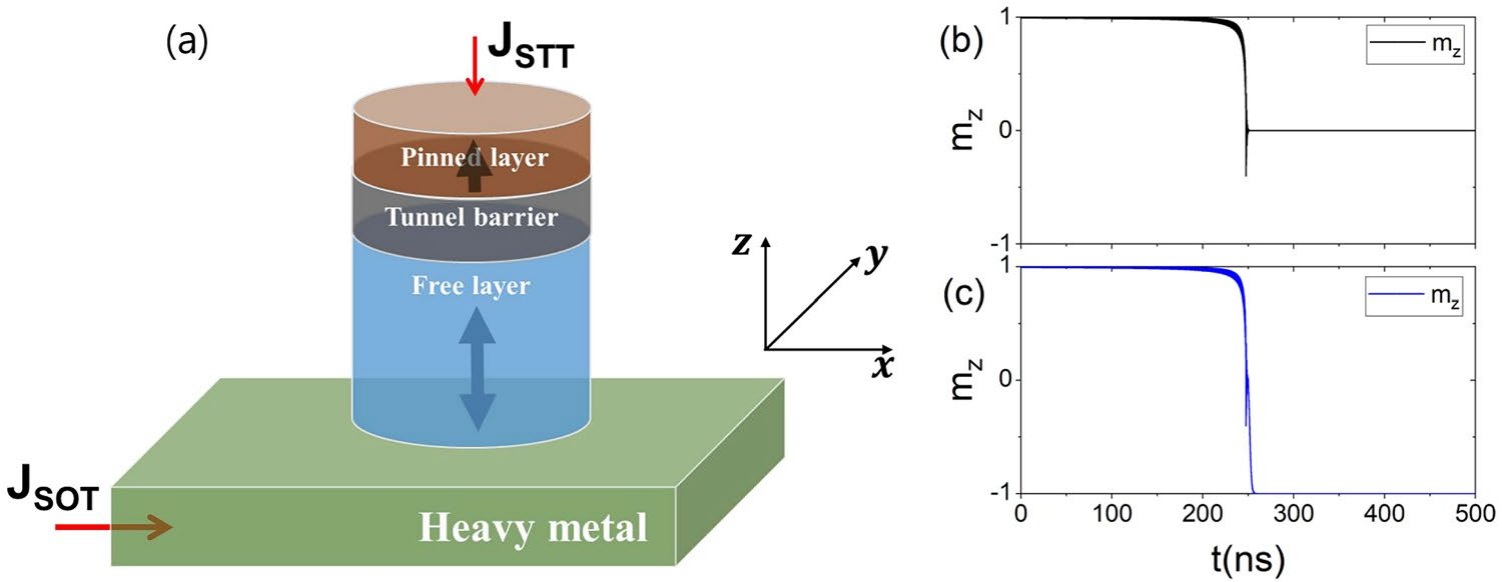
(**a**) Schematic of STT-SOT hybrid switching of MTJ with shape perpendicular magnetic anisotropy. The current flowing through the heavy metal (MTJ) induces the spin current by the spin-orbit effect (spin filtering effect). The combination of STT and SOT enables the deterministic switching of magnetization of the free layer. The method of STT-SOT hybrid switching of MTJ with S-PMA ensures the magnetization switching at lower current density and high thermal stability at sub-20 nm. (**b**,**c**) The magnetization dynamics of MTJ when $J_{\text {SOT}}=6.0\times 10^{12}$ $\text {A/m}^2$ and $J_{\text {STT}}=2.2\times 10^{11}$ $\text {A/m}^2$. (**b**) Continuous application of the current inducing the STT and SOT. (**c**) Turning off the current-induced SOT approximately 250 ns.

The magnetization switching of the MTJ with S-PMA through the interplay of the STT and SOT is depicted in Fig. 1a. The MTJ is integrated into the heavy metal (HM) for simultaneous application of STT and SOT to the MTJ. The current flowing through the HM induces an in-plane spin current originating from the spin-orbit effect, which exerts SOT on the magnetization of the MTJ free layer. On the other hand, the perpendicular spin current generated by the spin-filtering effect by the current flowing through the MTJ exerts STT on the magnetization of the MTJ free layer, where perpendicularly magnetized pinned layer is assumed.

The magnetization dynamics of the free layer is described by the LLG equation with additional STT and SOT terms^1^,

1 $$\begin{aligned} \frac{\partial {\vec{m}}}{\partial t} & = -\gamma {\vec{m}}\times \mathbf {H}_{\text {K,eff}} +\alpha \left( {\vec{m}}\times \frac{\partial {\vec{m}}}{\partial t}\right) -\gamma H_{\text {STT}}\left( {\vec{m}}\times {\vec{m}}_{\text {P}}\times {\vec{m}}\right) \nonumber \\ & \quad - \gamma H_{\text {SOT}}\left( {\vec{m}}\times \mathbf {\sigma }\times {\vec{m}}\right) - \beta \gamma H_{\text {SOT}}\left( {\vec{m}}\times \mathbf {\sigma }\right) , \end{aligned}$$

where ${\vec{m}}({\vec{m}}_P)$ is the magnetization unit vector of the free layer (pinned layer), $\mathbf {\sigma }$ is the unit vector of the spin moment generated by the spin-orbit effect, $\gamma$ is the gyromagnetic ratio, $\alpha$ is the Gilbert damping constant, $\beta$ is the spin-orbit field-like ratio. $\mathbf {H}_{\text {K,eff}}=(-\mu _{0}M_{S}N_{xx}m_{x} +H_{\text {Bulk}}m_{x}){\hat{x}}+(-\mu _{0}M_{S}N_{yy}m_{y} +H_{\text {Bulk}}m_{y}){\hat{y}}+(-\mu _{0}M_{S}N_{zz}m_{z}+H_{\text {I}}m_{z}){\hat{z}}$ is the effective magnetic field where $N_{xx}, N_{yy}$, and $N_{zz}$ are the demagnetization factor, $H_{\text {Bulk}}=2K_{\text {Bulk}}/M_{S}$ is the bulk anisotropy field, $H_{\text {I}}=2K_{\text {I}}t_{\text {f}}/M_{S}$ is the interfacial anisotropy field, $K_{\text {Bulk}}$ is the bulk anisotropy energy density, $K_{\text {I}}$ is the interfacial anisotropy energy density and $M_{S}$ is the saturation magnetization. $H_{\text {STT}}=\hbar \eta J_{\text {STT}}/2eM_{S}t_{\text {f}}$ and $H_{\text {SOT}}=\hbar \vartheta J_{\text {SOT}}/2eM_{S}t_{\text {f}}$ are the spin-transfer torque strength and spin-orbit torque strength, respectively, where $\hbar$ is Planck constant divided by $2\pi$, $\eta$ is the spin polarization efficiency, *e* is the electron charge, $\vartheta$ is the spin Hall angle, $J_{\text {STT}}$ is the current density flowing through the MTJ and $J_{\text {SOT}}$ is the current density flowing through the HM.

The critical switching current density of the MTJ with S-PMA can be obtained by employing the linearized LLG equation in the rotation coordinate, (*X*, *Y*, *Z*)^31,44^. The transformation matrix from the (*x*, *y*, *z*) coordinate to the (X, Y, Z) coordinates can be expressed as

2 $$\begin{aligned} {\mathbf {R}}= \left( \begin{array}{ccc} \cos \theta &{} 0 &{} -\sin \theta \\ 0 &{} 1 &{} 0 \\ \sin \theta &{} 0 &{} \cos \theta \end{array} \right) \left( \begin{array}{lll} \cos \varphi &{} -\sin \varphi &{} 0 \\ -\sin \varphi &{} \cos \varphi &{} 0 \\ 0 &{} 0 &{} 1 \end{array} \right) \end{aligned}$$

where $\theta$ and $\varphi$ are the polar and azimuthal angles, respectively. The relationship between the magnetization components of the (*x*, *y*, *z*) and the magnetization components of the (*X*, *Y*, *Z*) coordinates is expressed as follows through $\theta$ and $\varphi$: $m_{x}=m_{X}\cos \theta \cos \varphi -m_{Y}\sin \varphi +m_{Z}\sin \theta \cos \varphi$, $m_{y}=m_{X}\cos \theta \sin \varphi +m_{Y}\cos \varphi +m_{Z}\sin \theta \sin \varphi$, and $m_{z}=-m_{X}\sin \theta +m_{Z}\cos \theta$. In the equilibrium state, the magnetization has a small oscillation around the *Z*-axis, so it can be approximated by $m_{Z}\cong 1$ and $|m_{X}|, |m_{Y}|\ll 1$. The LLG equation in the (*X*, *Y*, *Z*) coordinate can be linearized as

3 $$\begin{aligned} \frac{1}{\gamma }\frac{d}{dt} \left( \begin{array}{ll} m_{X} \\ m_{Y} \end{array}\right) + {{\mathbf{M}}} \left( \begin{array}{ll} m_{X} \\ m_{Y} \end{array}\right) = {\mathbf {L}} \end{aligned}$$

where matrices ${{\mathbf{M}}}$ and ${\mathbf {L}}$ are $2\times 2$ and $2\times 1$, respectively. The components of matrix ${{\mathbf{M}}}$ are

4 $$\begin{aligned} \mathbf{M _\mathbf{11 }}= & {} \alpha (N_{\text {in}}\cos 2\theta -N_{z}\cos 2\theta ) -H_{\text {STT}}\cos \theta -H_{\text {SOT}}\sin \theta \sin \varphi +\alpha \beta H_{\text {SOT}}\sin \theta \sin \varphi \end{aligned}$$

5 $$\begin{aligned} \mathbf{M _\mathbf{12 }}= & {} (N_{\text {in}}\cos ^{2}{\theta }-N_{z}\cos ^{2}\theta ) +\alpha H_{\text {STT}}\cos \theta +\alpha H_{\text {SOT}}\sin \theta \sin \varphi +\beta H_{\text {SOT}}\sin \theta \sin \varphi \end{aligned}$$

6 $$\begin{aligned} \mathbf{M _\mathbf{21 }}= & {} -(N_{\text {in}}\cos 2\theta -N_{z}\cos 2\theta ) -\alpha H_{\text {STT}}\cos \theta -\alpha H_{\text {SOT}}\sin \theta \sin \varphi -\beta H_{\text {SOT}}\sin \theta \sin \varphi \end{aligned}$$

7 $$\begin{aligned} \mathbf{M _\mathbf{22 }}= & {} \alpha (N_{\text {in}}\cos ^{2}{\theta }-N_{z}\cos ^{2}\theta ) -H_{\text {STT}}\cos \theta -H_{\text {SOT}}\sin \theta \sin \varphi +\alpha \beta H_{\text {SOT}}\sin \theta \sin \varphi \end{aligned}$$

where $N_{\text {in}}=\mu _{0}M_{s}N_{yy}+H_{\text {Bulk}}$ and $N_{\text {z}}=\mu _{0}M_{s}N_{zz}-H_{\text {I}}$. Because an MTJ with a circular shape is considered, $N_{xx}=N_{yy}$. The magnetization instability is determined by $|m_{X}|,|m_{Y}|\propto \exp \{\gamma [\pm i\sqrt{\det [{{\mathbf{M}}}]-(\text {Tr}[{{\mathbf{M}}}]/2)^{2}} -\text {Tr}[{{\mathbf{M}}}]/2]t\}$, where $\det [{{\mathbf{M}}}]$ and $\text {Tr}[{{\mathbf{M}}}]$ are a determinant and trace of the matrix ${{\mathbf{M}}}$, respectively. The critical current density is derived when $\text {Tr}[{{\mathbf{M}}}]=0$ as follows:

8 $$\begin{aligned}{}&\alpha (N_{\text {in}}-N_{z})\cos 2\theta + \alpha (N_{\text {in}}-N_{z})\cos ^{2}\theta -2H_{\text {STT}}\cos \theta \nonumber \\&\quad -2H_{\text {SOT}}\sin \theta \sin \varphi +2\alpha \beta H_{\text {SOT}}\sin \theta \sin \varphi =0 \end{aligned}$$

The critical switching current density of the MTJ with S-MAM is obtained by combining Eq. (8) with the initial state^31^. The energy density with the spin-orbit field-like torque is

9 $$\begin{aligned} \varepsilon =-M_{S}\int d{\vec{m}}\cdot \vec {H}_{\text {K,eff}} -M_{S}\beta H_{\text {SOT}}\vec {m}\cdot {\hat{x}} \end{aligned}$$

The initial state can be obtained by minimizing the energy density. Then, it is expressed as

10 $$\begin{aligned} \theta= & {} \arcsin \frac{\beta H_{\text {SOT}}}{\mu _{0}M_{S}(N_{yy} -N_{zz})+H_{\text {Bulk}}-H_{\text {I}}} \end{aligned}$$

11 $$\begin{aligned} \varphi= & {} \frac{\pi }{2} \end{aligned}$$

The critical switching current density obtained by substituting Eqs. (10) and (11) into Eq. (8) is

12 $$\begin{aligned} J_{\text {STT,c}}=\frac{2eM_{S}t_{\text {f}}}{\hbar \eta } \frac{\alpha (\mu _{0}M_{S}(N_{yy}-N_{zz})+H_{\text {Bulk}} +H_{\text {I}})^2-0.5\alpha (\beta H_{\text {SOT}})^2 -\beta H_{\text {SOT}}^2}{\sqrt{(\mu _{0}M_{S}(N_{yy}-N_{zz}) +H_{\text {Bulk}}+H_{\text {I}})^2-(\beta H_{\text {SOT}})^2}} \end{aligned}$$

The magnetic parameters used are $K_{\text {Bulk}}=-110 \; \text {KJ/m}^3$, $K_{\text {I}}=2.2\times 10^{-6} \; \text {KJ/m}^2$, $M_{S}=1.2\times 10^{6} \;\text {A/m}$, $\alpha =0.005$, $\beta =2.0$, $\eta =0.4$ and $\vartheta =0.13$^17,23,41^. For comparison with the analytical estimation, macrospin calculation was performed at zero temperature. However, the thermal stability was calculated at $T=300$ K.

## Analytical and numerical results

We first investigate the thermal stability of the MTJ with shape anisotropy, interfacial anisotropy, and bulk anisotropy energies. Shape anisotropy energy is generated by magnetostatic interaction, and when $t_{\text {f}}<D$ ($t_{\text {f}}\ge D)$, it induces an anisotropy field in the in-plane (out-of-plane) direction. The interfacial anisotropy energy is induced between the ferromagnet and the MgO interface and aligns the magnetization in the out-of-plane direction. The voltage across the MTJ converts the interfacial anisotropy field from the out-of-plane to the in-plane direction. Considering the single-domain magnetization reversal and circular MTJ, the thermal stability, $\Delta$, is expressed as

13 $$\begin{aligned} \Delta =\frac{E_{\text {b}}}{k_{\text {B}}T}=\frac{\pi D^{2}}{4k_{\text {B}}T} \bigg [\frac{\mu _{0}M_{S}^{2}}{2}(N_{xx}-N_{zz})t_{\text {f}} +K_{\text {Bulk}}t_{\text {f}}+K_{\text {I}}\bigg ] \end{aligned}$$

where $E_{\text {B}}$ is the energy barrier between the two magnetic stable states, $k_{\text {B}}$ is the Boltzmann’s constant, *T* is the temperature, and $\mu _{0}$ is the vacuum permeability.

**Figure 2:**
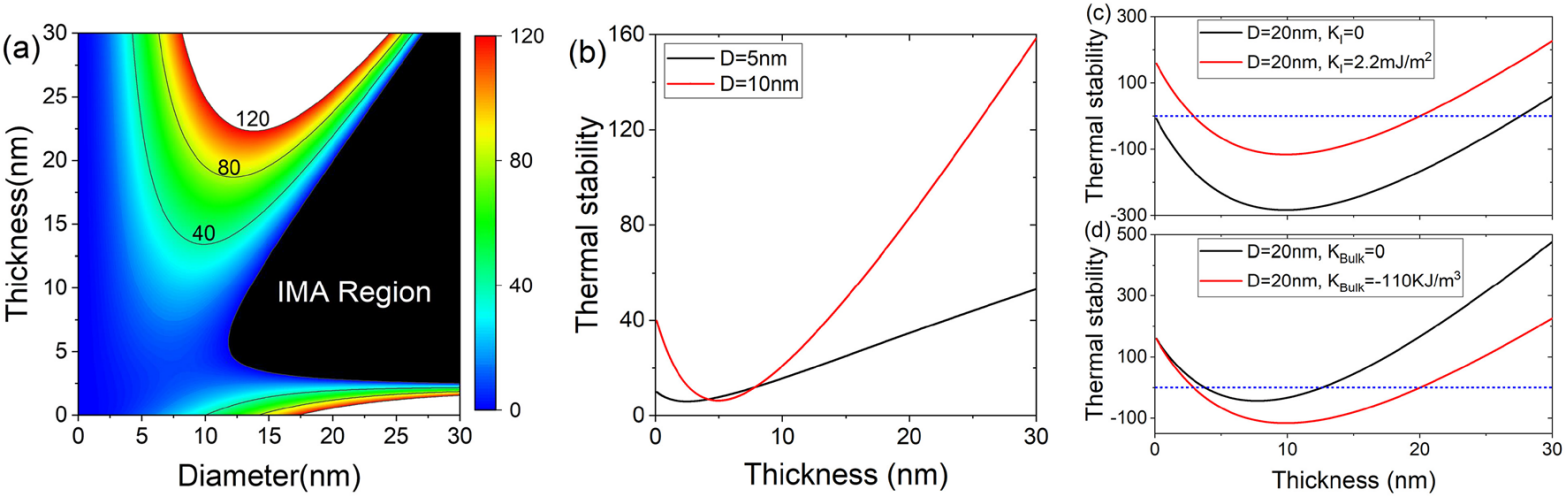
(**a**) Thermal stability as a function of thickness $t_{\text {f}}$ and diameter *D*. (**b**) Thermal stability as a function of $t_{\text {f}}$ for a given $D=5$ and 10 nm. (**c**) Comparison of thermal stability for $K_{\text {I}}=0$ and $K_{\text {I}}=2.2\times 10^{-3}$ $\text {A/m}^2$ for a given $D=20$ nm. (**d**) Comparison of thermal stability for $K_{\text {Bulk}}=0$ and $K_{\text {Bulk}}=-1.1\times 10^{5}$ $\text {A/m}^3$ for a given $D=20$ nm.

Figure 2a shows $\Delta$ as a function of thickness and diameter. Compared with Fig. 1a of a report by Watanabe^23^, the in-plane anisotropy region expands because of the influence of bulk anisotropy energy. Moreover, the contour of $\Delta$ line shifts upward. However, only the PMA region is exhibited for small diameters $(D\le 12$ nm). Figure 2b shows the thickness dependence of $\Delta$ for $D=5$ and 10 nm. In the I-PMA-dominant region, $\Delta$ decreases with increasing thickness, while in the S-PMA-dominant region, $\Delta$ increases as the thickness increases. The minimum value of $\Delta$ shifts to a large thickness with increasing *D* because of the movement of the S-PMA-dominant region as *D* changes. In Fig. 2c, $\Delta$ is compared at $K_{\text {I}}=0$ and $K_{\text {I}}=2.2\times 10^{-3}$ $\text {J/m}^{2}$. When $K_{\text {I}}=0$, the IMA is greatly expanded; even when the thickness is 30 nm, $\Delta$ is less than 80. This implies that, even in the S-PMA-dominant region, the I-PMA affects PMA, and to obtain a high $\Delta$ at a smaller thickness, one must consider a material with a large $K_{\text {I}}$. Figure 2d shows $\Delta$ at $K_{\text {Bulk}}=0$ and $K_{\text {Bulk}}=-1.1\times 10^{5}$ $\text {J/m}^3$. The boundary between the I-PMA and the IMA does not change significantly, while the boundary between the S-PMA and IMA changes significantly. Furthermore, in the S-PMA-dominant region, $\Delta$ decreases significantly at $K_{\text {Bulk}}=-1.1\times 10^{5}$ $\text {J/m}^3$ compared with $K_{\text {Bulk}}=0$. This implies that, to obtain a high $\Delta$ at a given diameter and thickness, one can consider a material with a small bulk anisotropy energy.

**Figure 3:**
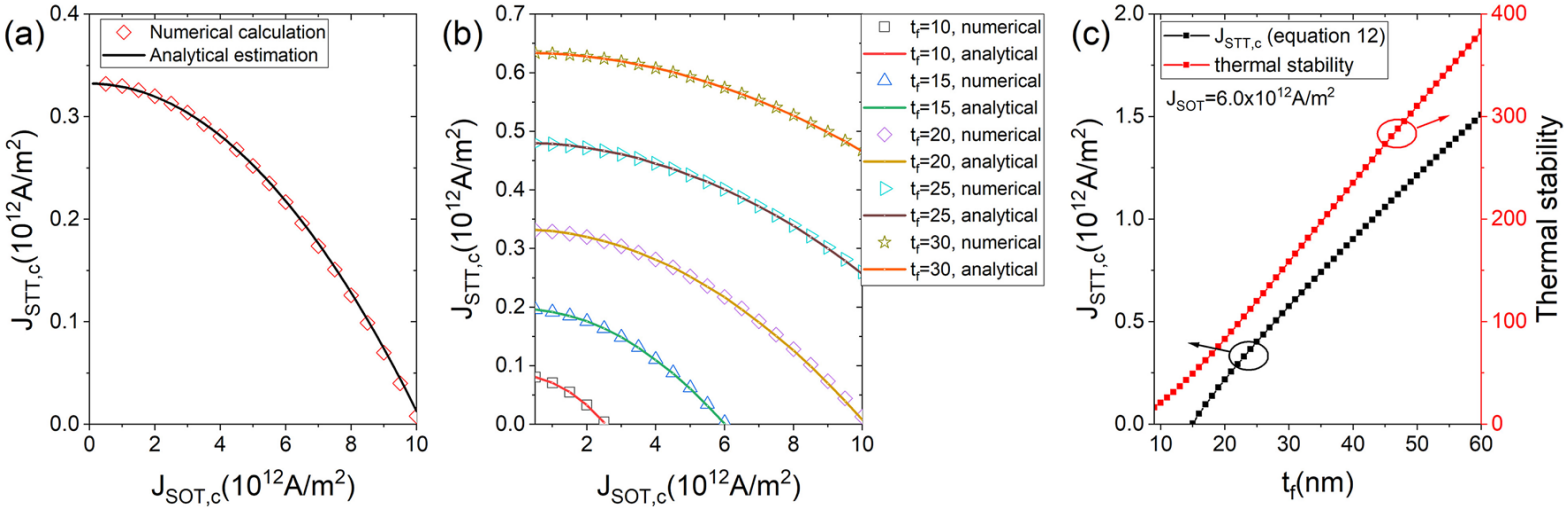
Analytical estimation and numerical calculation of the critical switching current density for a given $D=10$ nm. (**a**) $t_{\text {f}}=20$ nm. $J_{\text {STT,c}}$ decreases nonlinearly with increasing $J_{\text {SOT,c}}$. (**b**) $t_{\text {f}}=10, 15, 20, 25$ and 30 nm. As $t_{\text {f}}$ increases, $J_{\text {STT,c}}$ increases. (**c**) $J_{\text {SOT,c}}=6.0\times 10^{12}$ $\text {A/m}^2$. As $t_{\text {f}}$ increases, $J_{\text {STT,c}}$ increases because the barrier height between two stable state increases with increasing $t_{\text {f}}$, where black (red) dotted line indicates the critical switching current density (thermal stability).

The analytical formula of the critical switching current density—Eq. (12)—is obtained using the magnetization instability condition. In STT-SOT hybrid switching, the critical switching current density in Eq. (12) does not guarantee $180^{\circ }$ magnetization switching (i.e., deterministic switching), because SOT induces magnetization in the in-plane direction. However, experimentally, after applying the current pulse that induces STT and SOT, the magnetization is placed in-plane. Then the SOT pulse is blocked, and the magnetization achieves deterministic switching by the STT. Figure 1b,c show the dynamics of the $m_{z}$ at $J_{\text {SOT}}=6.0\times 10^{12}$ $\text {A/m}^{2}$ and $J_{\text {STT}}=2.2\times 10^{11}$ $\text {A/m}^{2}$ for a given $D=10$ nm and $t_{f}=20$ nm, where $J_{\text {STT}}=2.2\times 10^{11}$ $\text {A/m}^{2}$ is the critical switching current density. Figure 1b shows that the magnetization is located in-plane by the continuous application of SOT- and STT-induced currents. Meanwhile, Fig. 1c shows the achievement of deterministic magnetization switching by blocking the SOT-induced current pulse after the in-plane position of $m_{z}$. Therefore, the switching current density in Eq. (12) equals the deterministic switching current density. The dependence of the critical switching current density of STT $(J_{\text {STT,c}})$ on that SOT $(J_{\text {SOT,c}})$ for a given $t_{\text {f}}=20$ nm and $D=10$ nm is shown in Fig. 3a by comparing the analytical estimation and numerical calculations. The analytical and numerical results agree well. As $J_{\text {SOT,c}}$ increases, $J_{\text {STT,c}}$ decreases nonlinearly. This agrees with a report by Wang describing the STT-SOT hybrid switching of the MTJ with I-PMA^41^. At a high $J_{\text {SOT,c}}$, a low $J_{\text {STT,c}}$ prevents damage to MgO barrier caused by the high current density. Figure 3b shows the critical switching current density as a function of $J_{\text {SOT,c}}$ and $J_{\text {STT,c}}$ for a given $D=10$ nm and various $t_{\text {f}}$. As $t_{\text {f}}$ decreases, magnetization switching is achieved at lower $J_{\text {SOT,c}}$ and $J_{\text {STT,c}}$ because $\Delta$ increases with increasing $t_{\text {f}}$ as shown in Figs. 2a and 3c. Figure 3c shows the dependence of $J_{\text {STT,c}}$ on $t_{\text {f}}$ for a given $D=10$ nm and $J_{\text {STT,c}}=6.0\times 10^{12}$ $\text {A/m}^{2}$. Figure 3c provides the minimum value of $t_{\text {f}}$ for $\Delta \ge 80$

.

**Figure 4:**
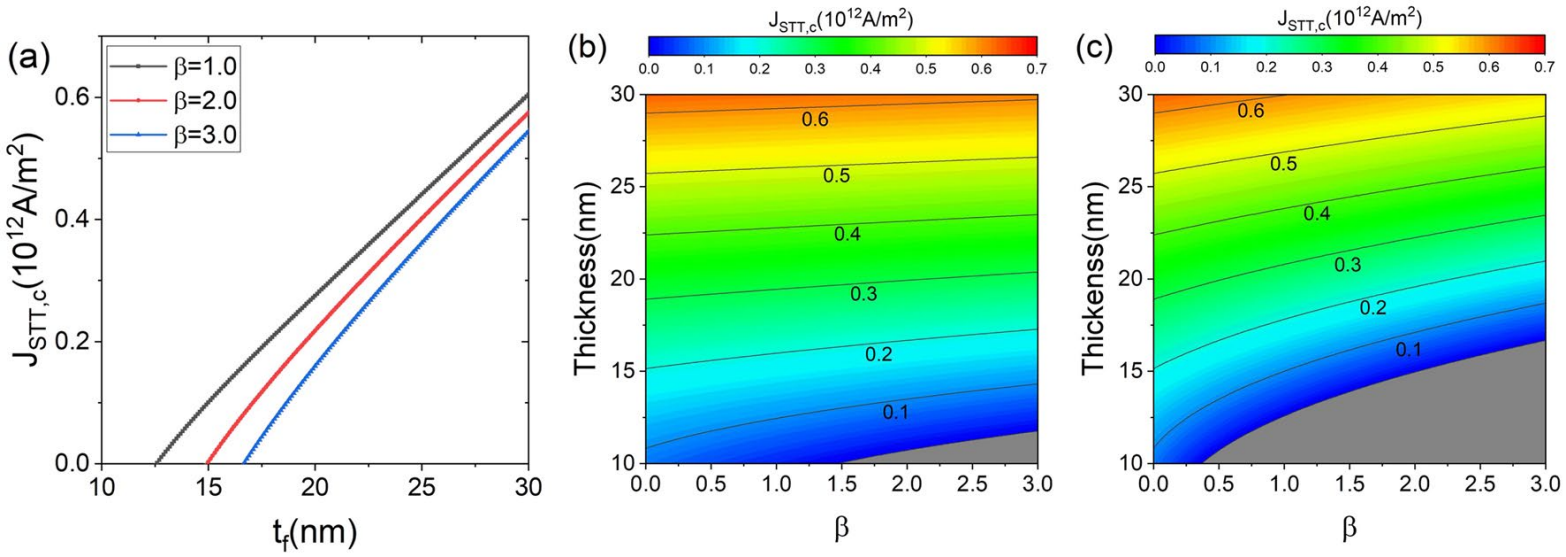
Thickness and spin-orbit field-like torque dependence of $J_{\text {STT,c}}$ for fixed diameter$=10$ nm. (**a**) $J_{\text {STT,c}}$ as a function of $t_{\text {f}}$ for different $\beta$ and fixed $J_{\text {SOT}}=6.0\times 10^{12}$ $\text {A/m}^{2}$. (**b**) and (**c**) $J_{\text {STT,c}}$ as a function of $t_{\text {f}}$ and $\beta$ for a given $J_{\text {SOT}}=3.0\times 10^{12}$ $\text {A/m}^{2}$ (**b**) and $J_{\text {SOT}}=6.0\times 10^{12}$ $\text {A/m}^{2}$ (**c**).

The critical switching current density is determined by the balance between the energy supplied and the dissipation resulting from damping. A positive spin-orbit field-like torque increases the energy supplied; consequently, the critical switching current density declineds^31^. On the other hand, an increase in the barrier height between two magnetic stable states with increasing thickness increases the critical switching current density. Figure 4 shows the dependence of $J_{\text {STT,c}}$ on thickness, and $\beta$. Figure 4a shows the $J_{\text {STT,c}}$ as a function of $t_{\text {f}}$ for different $\beta$ and fixed $J_{\text {SOT}}=6.0\times 10^{12}$ $\text {A/m}^2$. A lower $J_{\text {STT,c}}$ is exhibited at a higher $\beta$, as shown in Fig. 4a. The difference in $J_{\text {STT,c}}$ between different $\beta$ at lower thicknesses is greater than that at higher thicknesses. For example, at $t_{\text {f}}=17$ nm, the difference in $J_{\text {STT,c}}$ between $\beta =1$ and $\beta =3$ is $0.1541\times 10^{12}$ $\text {A/m}^2$, whereas, at $t_{\text {f}}=30$ nm, the difference in $J_{\text {STT,c}}$ between $\beta =1$ and $\beta =3$ is $0.06\times 10^{12}$ $\text {A/m}^2$. Figure 4b,c show the $J_{\text {STT,c}}$ in the plane of thickness and $\beta$ at $J_{\text {SOT}}=3.0\times 10^{12}$ $\text {A/m}^2$ and $J_{\text {SOT}}=6.0\times 10^{12}$ $\text {A/m}^2$, respectively, where the grey region of Fig. 4b,c is where in-plane switching is possible only by SOT without STT. In this region, deterministic switching can be achieved with a small current density flowing through the MTJ, inducing STT. Although $J_{\text {STT,c}}$ decreases (increases) with increasing $\beta$ $(t_\text {f})$, in the plane of $t_\text {f}$ and $\beta$, $J_{\text {STT,c}}$ increases slowly as $t_\text {f}$ and $\beta$ increase. The slope of $J_{\text {STT,c}}$ in Fig. 4c is steeper than that in Fig. 4b because the slope of $J_{\text {STT,c}}$ is steeper at higher $J_{\text {SOT}}$ (see Fig. 3a).

**Figure 5:**
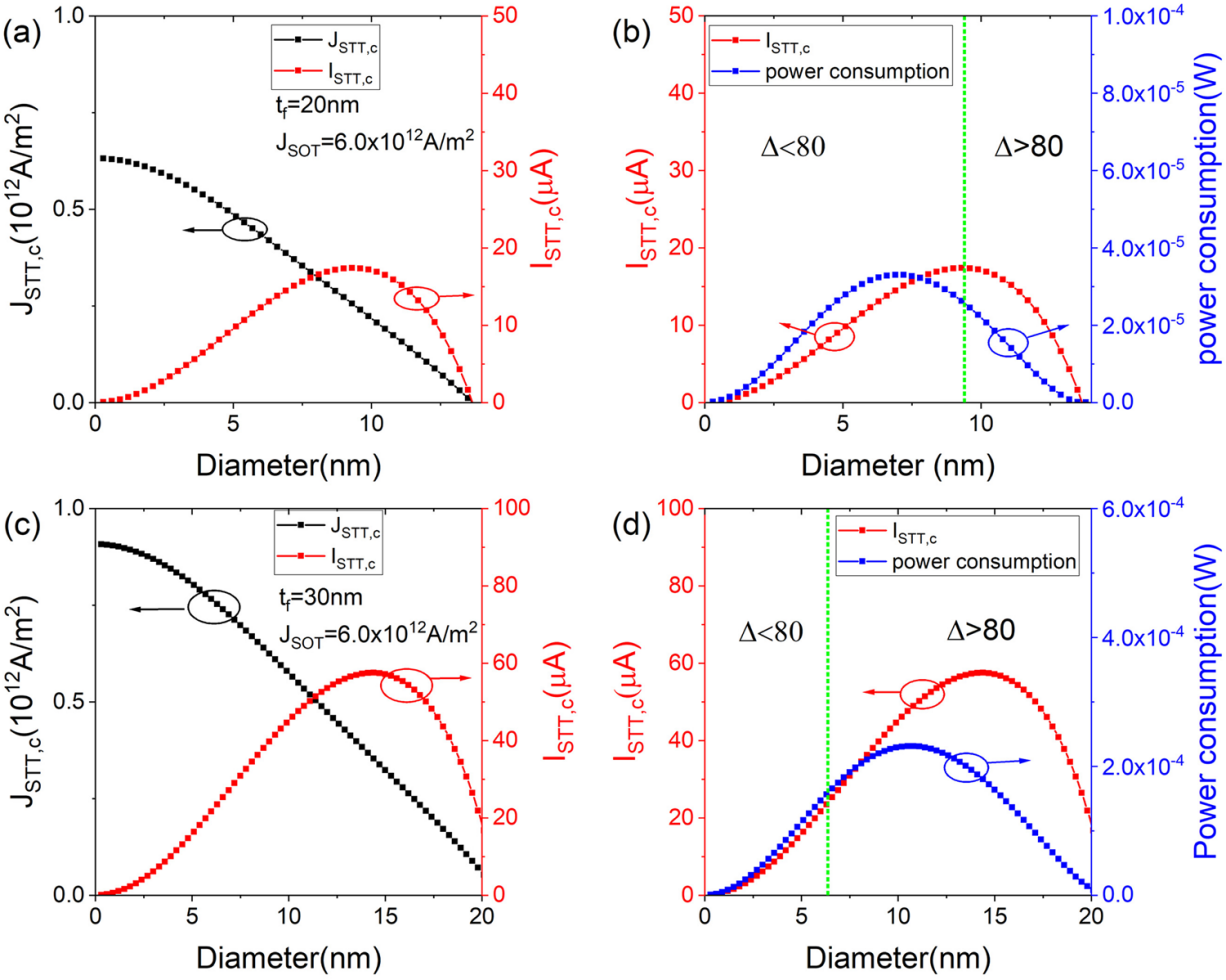
The diameter dependence of $J_{\text {STT,c}}$, $I_{\text {STT,c}}$ (**a**,**c**) and power consumption (**b**,**d**) at different $t_{\text {f}}=20$ nm (**a**,**b**) and $t_{\text {f}}=30$ nm (**c**,**d**) for $J_{\text {SOT}}=6.0\times 10^{12}$ $\text {A/m}^2$. The black, red and blue dotted lines represent $J_{\text {STT,c}}$, $I_{\text {STT,c}}$ and power consumption, respectively. The green dotted line indicates the boundary of $\Delta =80$.

The $J_{\text {STT,c}}$, $I_{\text {STT,c}}$ and power consumption by STT are investigated as a function of *D* for a given $t_{\text {f}}=20$ ($t_{\text {f}}=30$) nm and $J_{\text {SOT}}=6.0\times 10^{12}$ $\text {A/m}^{2}$ in Fig. 5a–d. As shown in Fig. 5a,c, $J_{\text {STT,c}}$ decreases monotonically with increasing *D*, whereas $I_{\text {STT,c}}$ decreases after increasing as *D* increases. The resistance of the MTJ is 50.12 (62.28) k$\Omega$ for a parallel (antiparallel) state for a given $t_{\text {f}}=15$ nm and $D=10$ nm^23,45^. The resistivity, $\rho =2.95\times 10^{-4}$ $\Omega \, \text{m}$, is obtained when using the average resistance. Employing the $\text {P}=I^{2}R$, the power consumption of the MTJ by STT is calculated as a function of *D* for a given $t_{\text {f}}=20$ and 30 nm, as shown in Fig. 5b,d, respectively. The energy consumption ($\Delta E$) can be obtained by multiplying power by pulse duration ($\Delta t_{\text {SW}}$). At $J_{\text {STT,c}}$, the magnetization switching time ($t_{\text {SW}}$) is very large. However, as the $J_{\text {STT}}$ increases, the switching time rapidly decreases (supplementary Information 3). Power consumption increases and then decreases as the diameter increases^17^. However, in the region of $\Delta \ge 80$, different patterns appear at the $t_{\text {f}}=20$ and 30 nm. Figure 5b shows that the power consumption decreases with increasing *D* for $\Delta \ge 80$. On the other hand, regions with $\Delta \ge 80$ are separated by the peak in Fig. 5d. In the left (right) region of the peak, the power consumption increases (decreases) with increasing *D*. This implies that using an MTJ with a smaller (larger) diameter is advantageous in terms of the energy efficiency in the region to the left (right) of the peak.

**Figure 6:**
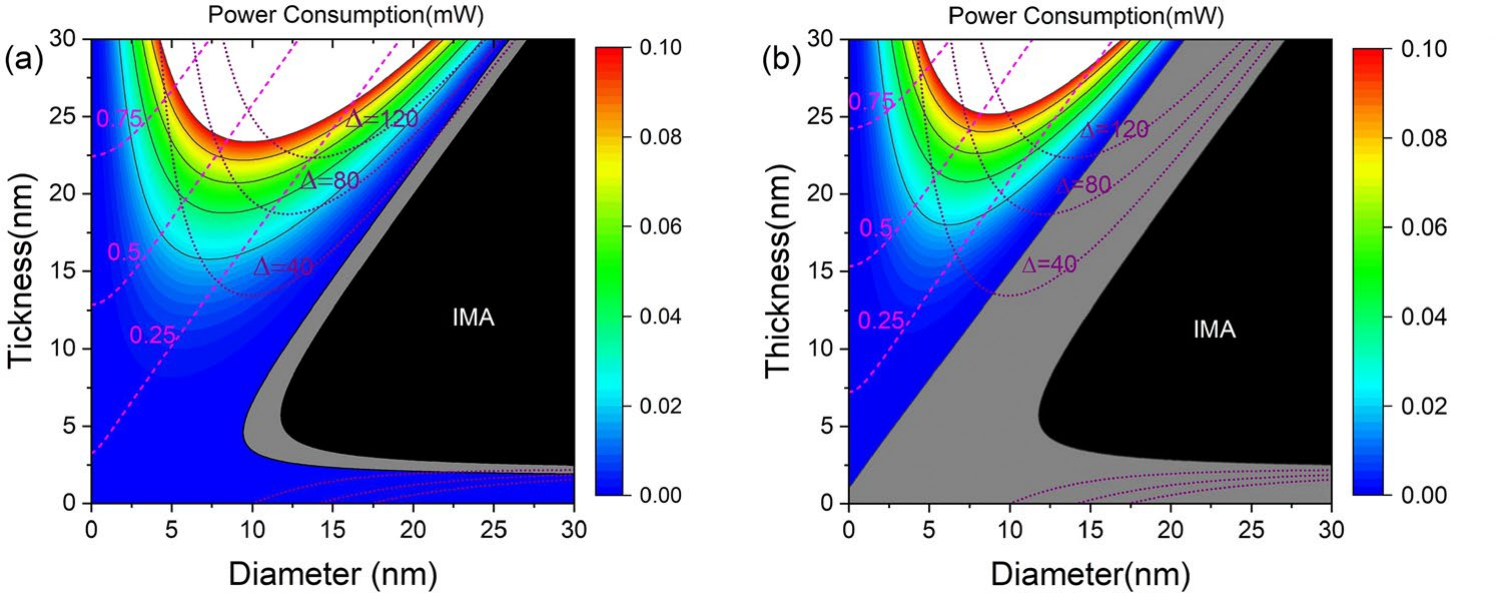
$J_{\text {STT,c}}$ and power consumption by STT as a function of thickness and diameter for $J_{\text {SOT}}=1.0\times 10^{12}$ $\text {A/m}^2$ (**a**) and $J_{\text {SOT}}=6.0\times 10^{12}$ $\text {A/m}^2$ (**b**), where the power consumption is obtained at critical switching current. The magenta dashed line and purple dotted line indicate $J_{\text {STT,c}}$ $(10^{12}\text {A/m}^2)$ and thermal stability, respectively.

Figure 6 shows the $J_{\text {STT,c}}$, thermal stability, and power consumption by the current inducing the STT as a function of thickness and diameter at $J_{\text {SOT}}=1.0\times 10^{12}$ and $J_{\text {SOT}}=6.0\times 10^{12}$ $\text {A/m}^2$, where the power consumption is obtained at the critical switching current. The grey region of Fig. 6a,b is the region where in-plane switching is possible only by SOT without STT. For a given diameter, the power consumption increases with increasing $t_{\text {f}}$ because of the increasing resistance of the MTJ and the barrier height between two magnetic stable states. For a given $t_{\text {f}}$, the power consumption increases and then decreases as the diameter increases. At the $\Delta =80$ contour line, the power consumption by the current inducing the STT is reduced as the diameter increases. In comparison with Fig. 6a,b, a pattern of increasing and then decreasing power consumption in the region of $\Delta \ge 80$ appears at a lower $t_{\text {f}}$ at low $J_{\text {SOT}}$, e.g., at $J_{\text {SOT}}=1.0\times 10^{12}$ $\text {A/m}^2$, the power consumption increases from $D=8.5$ to 9.4 nm and then is reduced as *D* increases at $t_{\text {f}}=22$ nm. However, at $J_{\text {SOT}}=6.0\times 10^{12}$ $\text {A/m}^2$, the power consumption decreases with increasing *D* at $t_{\text {f}}=22$ nm, but the power consumption increases from $D=7.8$ nm to $D=8.5$ nm at $t_{\text {f}}=24$ nm.

## Conclusion

The critical switching current density of MTJ with S-PMA through the interplay of STT and SOT was investigated through theoretical methods and macrospin simulation. The MTJ with S-PMA provides high thermal stability for the application of STT-SOT MRAM at diameters less than 20 nm, even in the presence of in-plane bulk anisotropy energy. Moreover, $J_{\text {STT,c}}$ decreases nonlinearly as $J_{\text {SOT,c}}$ increases. Furthermore, $J_{\text {STT,c}}$ increases slowly in the planes of $\beta$ and $t_{\text {f}}$, although increasing $\beta$ leads to a reduced $J_{\text {STT,c}}$. This is attributed to an increase in $t_{\text {f}}$, resulting in an increase in the barrier height between two magnetic stable states. This contributes to an increase in $J_{\text {STT,c}}$. Power consumption by STT at the critical switching current was investigated as a function of thickness and diameter. In the region of $\Delta \ge 80$, the power consumption reduces with increasing diameter at low $t_{\text {f}}$, while the power consumption increases and then decreases with increasing diameter at high $t_{\text {f}}$.

## Supplementary Information

Supplementary Information.
