# Supplementary material for: Critical switching current density of magnetic tunnel junction with shape perpendicular magnetic anisotropy through the combination of spin-transfer and spin-orbit torques
Source: Sci Rep. 2021 Nov 24;11:22842. doi: 10.1038/s41598-021-02185-3 (PMC8613283; doi:10.1038/s41598-021-02185-3)
Supplement: Supplementary file 1 — Supplementary Information. [file 41598_2021_2185_MOESM1_ESM.pdf]

# Supplementary Information

**Doo Hyung Kang**[1] **and Mincheol shin**[1]

[1] School of Electrical Engineering, Korea Advanced Institute of Science and Technology, 34141 Daejeon, Republic of Korea

E-mail: ppassionata@kaist.ac.kr, mshin@kaist.ac.kr

## 1. Influence of the thermal activation on the critical switching current density

In this section, the influence of the thermal field on the critical switching current density of the MTJ with S-PMA through the interplay of STT and SOT is investigated. Under the assumption of single domain switching[1], we investigate the critical switching current density using macrospin simulation. The thermal field added to the effective magnetic field of the LLG can be written as

$$\vec{H}_{\mathrm{th}} = \vec{G}\sqrt{\frac{2\alpha k_B T}{\gamma M_s V_f \Delta t}} \tag{S1}$$

where $\vec{G}$ is the vector created by a Gaussian random number generator, $k_B$ is the Boltzmann constant, $M_s$ is the saturation magnetization, $V_f$ is the volume of the free layer, and $\Delta t$ is a small amount of time corresponding with the time step in the numerical calculation.

Sfig. 1 shows the critical switching current density for $t_{\mathrm{f}} = 20$ nm and $D = 10$ nm in presence of thermal field. In Sfig. 1(a), we show $m_z$ dynamics for $J_{\mathrm{SOT}} = 6.0 \times 10^{12}$ A/m$^2$. The critical switching current density is observed at $J_{\mathrm{STT}} = 1.98 \times 10^{11}$ A/m$^2$, where $m_z$ is located in-plane by the simultaneous application of SOT- and STT-induced currents. The influence of the thermal field on the critical switching current density is shown in Sfig. 2(b). In presence of the thermal field, the critical switching current density decreases.

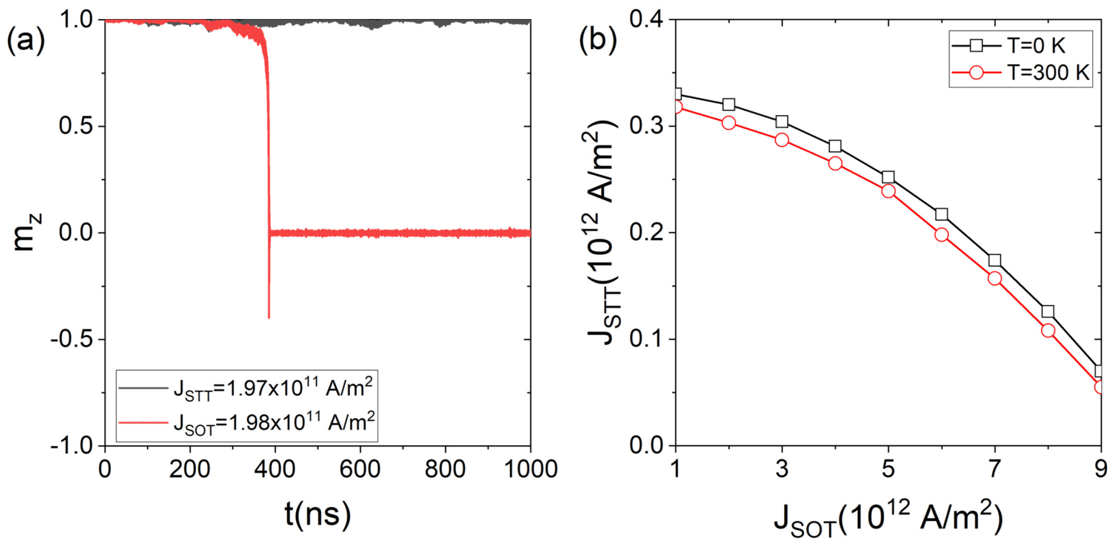


**Sfigure 1.** (a) Magnetization dynamics for $J_{\mathrm{SOT}} = 6.0 \times 10^{12}$A/m$^2$, and various $J_{\mathrm{STT}}$ (b) Comparison of Critical switching current density at $T = 0$ K and $T = 300$ K.

## 2. Comparison of power consumption in STT switching, SOT switching, and STT-SOT hybrid switching

Power consumption of conventional STT switching, SOT switching, and STT-SOT hybrid switching by STT is investigated at the critical switching current density in this section. We consider Ta as a heavy metal. The parameters used are $\rho_{\text{Ta}} = 1.9 \times 10^{-6}$ $\Omega \cdot m$, $L_{\text{Ta}} = 20$ nm, $W_{\text{Ta}} = 20$ nm, and $t_{\text{Ta}} = 2$ nm, where $\rho_{\text{Ta}}$ is the resistivity of Ta, $L_{\text{Ta}}$ is the length of Ta, $W_{\text{Ta}}$ is the width of Ta, and $t_{\text{Ta}}$ is the thickness of Ta. The Other parameters used are the same as those used in main text. The critical current denstiy of STT switching and SOT switching can be expressed as[2, 3]

$$J_{\text{STT,sw}} = \frac{2eM_S t_{\text{f}}\alpha}{\hbar\eta}(\mu_0 M_S(N_{yy} - N_{zz}) + H_{\text{Bulk}} + H_{\text{I}}), \tag{S2}$$

$$J_{\text{SOT,sw}} = \frac{2eM_S t_{\text{f}}}{\hbar\vartheta}\frac{\sqrt{2\alpha}(\mu_0 M_S(N_{yy} - N_{zz}) + H_{\text{Bulk}} + H_{\text{I}})}{\sqrt{\beta(2+\alpha\beta)}} \tag{S3}$$

For SOT switching, the critical switching current density is large because it is less dependent on Gilbert damping compared to STT switching. Therefore, the power consumption of SOT switching is large. However, as the diameter increases, the power consumption of SOT switching decreases sharply as shown in Sfig. 2.

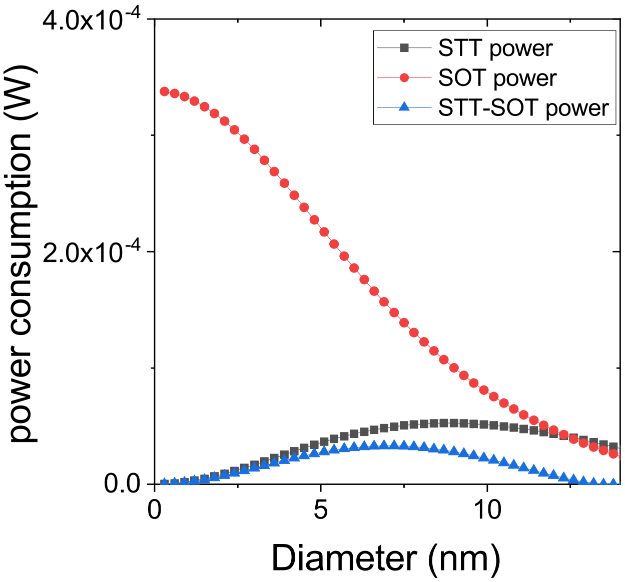


**Sfigure 2.** Power consumption of STT switching, SOT switching, and STT-SOT switching by STT.

## 3. Switching time of STT-SOT hybrid swithcing in MTJ with S-PMA

In this section, we investigate the switching time ($t_{\rm sw}$) of MTJ with S-PMA through the interplay STT and SOT as a function of $J_{\rm STT}$. When $J_{\rm SOT}$ and $J_{\rm STT}$ above the critical switching current density are applied simultaneously, $m_z$ is located in the $xy$ plane or $m_z = -1$. Therefore, in numerical calculation, magnetization switching is defined as

$$\lim_{t\to\infty} |m_z(t) - m_z(0)| \geq 0.9 \tag{S4}$$

Sfig. 3 shows the $t_{\rm sw}$ of the MTJ with S-PMA for a given $J_{\rm SOT} = 6.0 \times 10^{12}$ A/m$^2$, $t_{\rm f} = 20$ nm, and $D = 10$ nm. In Sfig. 3(a), we observe the achievement of deterministic magnetization switching. Here $J_{\rm SOT}$ is turned off when $|m_z(t) - m_z(0)| \geq 0.9$. In Sfig. 3(b) shows the $t_{\rm sw}$ as a function of $J_{\rm STT}$. At the critical switching current density, $t_{\rm sw}$ is as large as about 250 ns and $t_{\rm sw}$ decreases significantly at $J_{\rm STT} = 3.0 \times 10^{11}$ A/m$^2$. And then $t_{\rm sw}$ slowly decreases with increasing $J_{\rm STT}$.

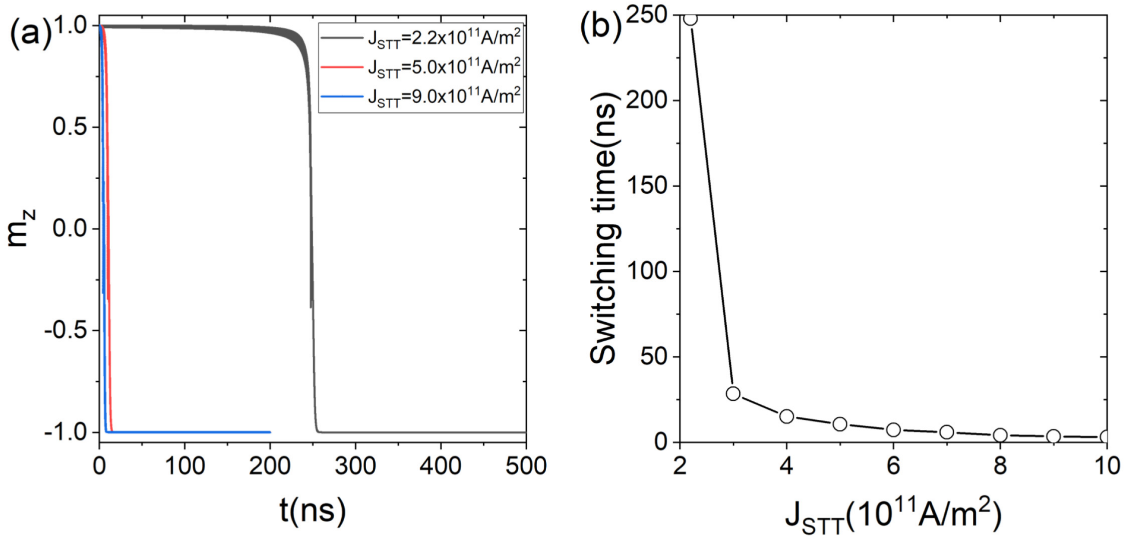


**Sfigure 3.** (a) Magnetization dynamics for $J_{\rm SOT} = 6.0 \times 10^{12}$A/m$^2$ and various $J_{\rm STT}$ (b) The switching time of the MTJ with S-PMA as a function of $J_{\rm STT}$ for $J_{\rm SOT} = 6.0 \times 10^{12}$A/m$^2$.

[1] Perrissin, N. et al. A highly thermally stable sub-20 nm magnetic random-access memory based on perpendicular shape anisotropy. *Nanoscale* **10**, 12187 (2018)

[2] Lee, K., Lee, S., Min, B. and Lee, K. Threshold current for switching of a perpendicular magnetic layer induced by spin Hall effect. *Appl. Phys. Lett.* **102**, 112410 (2013).

[3] Taniguchi, T., Mitani, S. and Hayashi, M. Critical current destabilizing perpendicular magnetization by spin Hall effect. *Phys. Rev. B* **92**, 024428 (2015).
